# Talent identification and development in an English Premiership rugby union club: the perspectives of players and coaches

**DOI:** 10.3389/fspor.2023.1120033

**Published:** 2023-11-06

**Authors:** Francesco Dimundo, Matthew Cole, Richard C. Blagrove, Jordan D. Herbison, Jennifer Turnnidge, Kevin Till, Francesca Vitali, Adam L. Kelly

**Affiliations:** ^1^ Department of Sport and Exercise, Research Centre for Life and Sport Sciences (CLaSS), School of Health Sciences, Birmingham City University, Birmingham, United Kingdom; ^2^ Department of Sport Science, Worcester Warriors Rugby Football Club, Worcester, United Kingdom; ^3^ Department of Research and Innovation, Italian Strength & Conditioning Society (ITASCS), Roma, Italy; ^4^ School of Sport, Exercise, and Health Sciences, Loughborough University, Loughborough, United Kingdom; ^5^ Department of Kinesiology & Physical Education, McGill University, Montréal, QC, Canada; ^6^ School of Kinesiology and Health Studies, Queen’s University, Kingston, ON, Canada; ^7^ Carnegie School of Sport, Leeds Beckett University, Leeds, United Kingdom; ^8^ Department of Neurosciences, Biomedicine and Movement Sciences, University of Verona, Verona, Italy

**Keywords:** qualitative, long-term athlete development, multidimensional analysis, sport psychology, talent identification and development, environmental constraints, rugby union

## Abstract

The pathway towards senior professional status in sport is affected by a multitude of factors. An abductive examination of the talent identification and development processes at an English Premiership rugby union (RU) club was undertaken for the present study. *Part one* examined the perspectives on the selection and development processes of senior academy male players (*n *= 8), whereas *part two* explored the perceptions of male coaches (*n *= 7). A total of three focus groups were used. Three main themes were confirmed by players and coaches: (a) task constraints, (b) performer constraints, and (c) environmental constraints. Specifically, although athletes and coaches believed that performer constraints were highly impactful on players' career in RU, there were inconsistencies surrounding the task and environmental constraints. Despite an indication that three common themes impacted an players path, this preliminary study shows an imbalance in the understanding of some of the key factors perceived to be important for talent progression in the present rugby academy. More research using similar qualitative methods is recommended to better understand the differences in opinions between players and coaches. Meanwhile, practitioners should consider implementing objective and holistic strategies to improve the talent pathway in English RU academies.

## Introduction

Becoming a senior professional athlete is the ambition of many youth participating in organised youth sports. Several authors have attempted to provide guidelines to optimise the Talent Identification (TID) and Talent Development (TD) paths to support this journey (e.g., 1). Although the two terms are used interchangeably, TID is defined as the process of recognising current participants with the potential to excel in a particular sport, whereas TD is the process of providing the most appropriate learning environment to realise this potential (2). Research examining TID and TD in sport indicates professional playing status is affected by a multitude of factors (1, 3, 4). However, the possible drawbacks of TID and TD systems have led key stakeholders (e.g., academy staff, players, players' parents/guardians) to question the efficacy and optimal recipe of these strategies (5).

The increasing popularity of Rugby Union (RU) has led to large financial investment in TID and TD systems from World Rugby Union (6) and national governing bodies (e.g. England Rugby) (7, 8). Such investments impact different areas of training and performance (e.g., physical, technical, tactical) among aspiring youth sport participants. For example, English Premiership RU's TID and TD systems follow a *wide and emergent* (e.g., offer a broad range of developmental opportunities and a focus on platers remaining in their own environment until adolescence) and *narrow and focused* (e.g., identify players with an individualised focused programme for long-term development) framework. The English system is managed by fourteen regional academies aligned to the respective professional RU clubs (7, 8). A recent systematic review of TID and TD in RU (9), which included studies conducted on English RU clubs, underscored the holistic nature of these processes. More specifically, Dimundo et al. (9) found that, independent of playing position, those academy players who progressed towards senior professional status possessed greater (a) body mass, (b) physical strength, power, and speed, (c) technical and tactical skill (e.g., advanced passing and catching accuracy, collective effectiveness), (d) psychological resources (e.g., resilience, cohesiveness, coping skills, and determination), and (e) socio-economic advantage (e.g., received higher support by coaches, clubs, schools, and local culture). Moreover, recent findings on relative age effect in RU found that birth quartile and socio-cultural context have a different impact upon progression in different playing position (10). Despite the growing body of literature exploring TID and TD in RU, further examination of players' and coaches' perceptions of TID and TD processes would advance understanding about how these processes unfold (11). In particular, there is the need to advance knowledge on specific factors that cannot emerge with quantitative procedures.

Examining TID and TD systems using qualitative methodological approaches can complement the quantitative approaches predominantly used in previous research (e.g., 9, 12, 13). Despite the growing body of literature exploring TID and TD in RU (e.g., 9), further examination of players’ and coaches' perceptions of TID and TD processes is required to better understand both selectors' and selected' perspectives (11). It is well known that TID and TD in sport should be analysed using diverse methodological perspectives since outcomes are affected by objective (e.g., measurable data from tests), and subjective (e.g., how players perceive sport situations and decisions (14), and interpretations of events (15). The most connected populations in an academy setup, and in a certain sense, the main *actors on the talent stage*, are those acting as “selectors” (i.e., coaches) and “selected” (i.e., players). Notably, the few qualitative studies exploring the TID and TD systems have focused on the coaches' perspective (16–18). For example, Chiwaridzo et al. (16) examined the anthropometrical, physical, technical-tactical, and psychological characteristics that Zimbabwean coaches perceived important for the TID and TD in adolescent RU school players. They found that coaches considered a range of fundamental qualities (e.g., technical, psychological, physical) underpinning the above-mentioned areas as decisive factors for players progression. Furthermore, Hill et al. (17) interviewed English Premiership RU coaches on psychological traits requested for players and found that a range of positive (e.g., self-regulated learning strategies, ownership and independence, motivation), negative (e.g., lack of commitment, lack of development awareness, mental health issues), and dual-effect (e.g., perfectionism, obsessive passion, over-commitment) characteristics were identified as essential for academy players' progression towards senior professional level. Additionally, Roberts and Fairclough (18) explored English regional development officer who were responsible for TID and TD processes awareness of relative age effects in RU. Surprisingly, though, the interviewed group revealed a general lack of knowledge, understanding, and awareness of relative age effects. Thus, implications from these studies remain limited to certain analysed factors and represent only the coaches' perspective, whist replication studies are also required to validate such findings.

The advantage of using a qualitative approach in this field of research is twofold. First, it can draw from different key stakeholders' perspectives, to hear the participants' voices while exploring emerging topics more in-depth, as well as help to inform researchers and practitioners on planning, practice, and decision-making (19, 20), with evidence that such themes cannot be captured using quantitative methods [e.g., player profiling, testing, questionnaires; (21)]. Second, analysing members of the same team, diverse and various approaches or policies that characterise various population of different clubs are eliminated, thus specific approaches and policies can be examined in depth. Therefore, consistent results can be explored on how perceptions of talent pathways differ depending on role. Furthermore, it has been reported that the perspectives of the two main samples from the TID and TD processes in sport academies (i.e., players *and* coaches) rarely feature in the literature to understand how organisational approaches actually affect TID and TD, despite their ability to offer valuable insights into the transition to the professional level (22). In addition to the paucity of qualitative research on TID and TD in RU, the current investigations only focus on the perspectives of coaches, leaving unanswered questions surrounding what players perceive important for selection and development in professional RU settings. As such, the aim of this paper was to evaluate the perspectives of both male academy players [under-21 (U21)] and academy coaches on the TID and TD processes in an English Premiership RU club.

## Methods

### Philosophical assumptions

The research team adopted a constructivist paradigm to conduct this study (23–26). Underpinning the study's purpose was the goal of understanding the meanings that players and coaches attach to their experiences within their RU club in relation to TID and TD. Given our interest in different perspectives, the research team's position rejects the notion of a single, “objective” reality. Instead, the reality of TID and TD in the RU club was evaluated as the cumulative experiences of each participant, interpreted by the individual, other team members in the respective focus group, and the research team [i.e., ontological relativism; (26)]. The research team recognised and considered our own subjectivities and how these influence the knowledge and theories used to design the study, evaluate the data, and disseminate the results. Further, the dynamic and transactional nature of the focus group method used to share knowledge amongst participants and members of the research team was informed by each individuals' personal experiences [i.e., subjective and transactional epistemology; (24)].

### Participants

Eight U21 male academy players (Table 1) and seven academy male coaches (Table 2) took part to this study.

**Table 1:** Shows players’ groups characteristics.

	Player group 1 (PG1)	Player group 2 (PG2)
Forwards (*n*)	2	1
Backs (*n*)	2	3
Age (M ± SD) (years)	19.9 ± 0.8	19.7 ± 0.8
RU playing experience (M ± SD) (years)	4.0 ± 0.8	5.7 ± 0.5
Age started playing RU (M ± SD) (years)	4.7 ± 0.5	8.0 ± 3.1

**Table 2:** Shows coaches’ group characteristics.

Coaches groups (CG)	
U15 coaches (*n*)	2
U16 coaches (*n*)	2
U18 and U21 coaches (*n*)	3
Rugby football union coaching (RFU) level 2 (*n*)	3
RFU level 3 (*n*)	2
RFU level 4 (*n*)	2
Age (M ± SD) (years)	37.4 ± 9.5
RU coaching experience (M ± SD) (years)	5.0 ± 3.6

### Procedure and study design

Institutional research ethics board approval was granted by (institution name blinded for review process) prior to data collection. Participants reviewed a letter of information and provided informed consent form prior data collection. The data collection was comprised of two parts in order to collect and analyse the data from both players and coaches' separately. All group discussions were organised by the lead author on three different days across two consecutive weeks in February 2020. Each focus group lasted approximately 60-minutes and was held in a meeting room at the club training ground, which had a comfortable setting that encouraged open discussion and interaction. Holding the focus groups at the club's facility was convenient and allowed participants to feel in a familiar setting and in a space where they were more comfortable to share information. Each focus group conversation was recorded by two video cameras (Sony HDR-CX240E Handycam) and two microphones (7RYMS RimoMic Lite LN Mini) in order to collect details of the discussions, and facilitate subsequent anonymised transcription of dialogue. While data collection was set by the first author, the conversation was facilitated by the first author for PG1 and PG2, and by the fifth author for the CG. Other authors (*initials blinded for review*) were also present during the focus groups to assist with recording equipment.

All focus groups followed a semi-structured design, whereby participants had the opportunity to discuss and reflect upon their experiences within an organised, yet flexible structure (21). During the focus groups discussions, the facilitator helped the flow of the conversation by encouraging participants to develop on initial interactions and promoting responses that reflected participants' perspectives on TID and TD processes in their RU academy (e.g., “How would you describe what role a coach plays in facilitating athlete development?”, “What do you think is important to take into account when developing a player?”). It was emphasised throughout the focus groups that there were no “correct” or “incorrect” answers to the questions and that confidentiality would be preserved.

### Data analysis

An adapted version of the abductive thematic analysis (27, 28) was used to explore the phenomenon of TID and TD from the perspectives of coach and athlete members of an English Premiership RU club. This approach, has been largely adopted in sport literature (e.g., 29) and has recommended when analysing data concerning multifaceted topics (e.g., 30–37). This analysis enables information to be shaped abductively (i.e., confirming existing theories from the data), which is a strategy that has been found important in qualitative research since it provides the development of theories based on empirical data collection in a specific context (33). The *specific lens* through which the data was analysed as part of the abductive approach was the constraints based on the ecological dynamics framework, which has been applied to previous studies on TID and TD (4, 9). The ecological dynamic framework is a concept emphasized by the “*constraints-based approach*” described by Davids (38), reflects a topic previously shaped by several authors (e.g., 39, 40), and uses as theoretical platform Bronfenbrenner (41), Newell (42), and Newell et al. (43) works on *constraints* and *ecological system approach*.

Four main steps were followed and adapted from previous literature (36, 37, 44), including: (a) recording and transcribing focus groups, (b) creating codes, (c) defining and merging codes and categories, and (d) refining themes. Specifically, this process included: (a) finding holistic theories in TID and TD in sport, (b) creating tags, (c) creating sub-categories, (d) creating categories, and (e) refining themes. Before refining final themes, discussion topics were organised into categories and sub-categories. The first stage consisted in recording and transcribing the focus groups using NVivo 12 (QSR International, Melbourne, Australia). The second stage consisted in identifying basic conceptual units called “meaning units”. These were established based on part of the text that contained one idea that was coded with a descriptive name (45). Tags were created with words containing the meaning units and were “flagged” when important information was established. NVivo enabled to label meaning unit that could have been easily search for content check by authors and eventually replaced with adequate modification. The third stage consisted in the creation of “sub-categories” involving a higher level of tags, which comprised a similar type of description to the initial level of analysis. Where there were similarities across each sub-category, tags were assembled into “categories”, representing a higher level of inspection. The software enabled to quantify contents in order to number each category, which was useful for authors during final interpretation of data. Finally, further analysis of the data consisted of merging similarities between flagged groups to determine “themes”, whereby relationships were identified and organised into higher-order groups. All themes were then independently reviewed before being agreed by the research team. Then, participants confirmed that all information and themes reported were correct. Anonymised example quotations have been provided throughout the results. A similar structure for analysis and reporting of data has been used previously in sport literature (e.g., 16, 46).

### Establishing trustworthiness and methodological rigor

The aim of qualitative research is not to produce replicability in the same way as quantitative research, but (as also for the quantitative approach) to provide results that are consistent with the data collected (47). Rigor was improved by the insider perspective of the lead author (i.e., they were working as strength and conditioning coach for the respective club) of this study, whereas the other team members and co-authors offered a more outsider perspective (i.e., they were professionals not working for the club) during the analysis process. Additionally, to enhance the rigor of the research, it is important to highlight that the lead facilitators were skilled in working with, communicating with, listening to, and understanding young athletes and professional coaches. Indeed, these skills that have been previously identified as being useful for engaging dialogues with specific populations (48). The research lead also had an extensive knowledge of the team, coaches, and players at the RU club, which promoted access to participants and facilitated the flow of the discussions.

This research was part of a 3 year exploration on the topic of TID and TD processes authorised by an English Premiership RU club. Therefore, this qualitative work, which is the first on TID and TD in RU that has analysed both players and coaches, was considered a *worthy topic*. The use of two independent focus groups for the players and a separate one for the coaches provided both similarities and contrasting perspectives on the selection and development processes in one of the few English Premiership RU academies, which ensured that the study met the criteria for *rich rigor*. The criterion of s*incerity* was encompassed throughout all the steps of the research process, whereby each author avoided bias during data collection and analysis. *Credibility* in the present study was met through the accuracy of the reported data and the reflections from all participants, whilst following and adapting widely used thematic guidelines (44, 49, 50) alongside using contemporary transcription and coding software (i.e., NVivo 12). The nature of the present findings embraced the criterion of *resonance* since they recalled the perceptions of academy players within the RU environment, which could be familiar to readers. Moreover, this investigation represents an attempt in advancing practitioners' knowledge on the topic of TID and TD in RU, which could provide a *significant contribution* to the applied sport science field as well as offer replication study in some areas (e.g., coaches perspectives) to validate previous findings. Since this piece of work received both organisational and institutional ethical approval from their respective administrative and ethics board, adequate *ethical* procedures were always followed. Finally, to ensure *meaningful coherence*, the research group believed this study achieved its stated goals and interconnected each stage of the research process so as to accomplish the intended outcomes. These considerations are based on the criteria suggested when analysing qualitative data (51).

## Results

### Part one: players' focus groups

Table 3 reports the PG1 and PG2 results and provides additional examples of relevant quotations for each of the categories identified. It should be noted that since it was not possible to report examples of quotations for each of the sub-categories, some quotes are duplicated in Table 3 to offer a complete overview. Conceptualisation of the raw-data sub-categories revealed eleven categories in total: (a) sport participation history, (b) activity type, (c) game exposure, (d) anthropometric, (e) physiological, (f) psychological and psychosocial, (g) technical-tactical, (h) national, (i) socio-economic, (j) family, and (k) culture. Finally, these lower-order themes were categorised into the three higher-order themes that were consistent with the constraints based on the ecological dynamics framework. including: task, performer, and environmental. According to each thematic description, TID and TD paths seem influenced by a multitude of factors that impact professional players’ trajectory in unison. Specifically, these themes aligned with constraints described in previous works on TID and TD in sport (4, 9) and add value to the existing theory of the ecological dynamic theory (for an in depth discussion of this framework, please see early works of (40, 42). As such, the following results are presented using these higher order themes.

**Table 3:** Players’ focus groups results.

Theme	Categories	Sub-categories	Categories’ additional example quotation
Task constraints	Sport participation history	Multisport background	“I preferred football up until I was 11, probably, then changed to rugby. And cricket, I preferred cricket until […] I started enjoying rugby and I started rugby. And a bit of athletics as well, on the side”. PG1
	Activity type	Exposure to training Consistency in good performance Position specific requirements Repetition of skills Dedication to improve skills Enjoy the skills Handling skills Skill transferability Skill-set variety	“[…] It's then what people are actually doing away from training and away from the club. […] So, it's those players who do that extra training bit, who have that extra want to not lose that are the ones that end up coming through and out the other end”. PG1
	Game exposure	Game played	“Because I think the more you're out on the pitch, the better you get as a player”. PG2
Performer constraints	Anthropometric	Height	“Because I feel like rugby is not that specialised, i.e., you've got to have loads of different qualities about yourself a part being tall”. PG2
	Physiological	Multitude of physical factors Individual characteristics Speed Fitness Strength Agility Physicality Work rate Jump qualities Quickness	“Like you've got to have speed, be quick, you've got to be agile”. PG2
	Psychological and psychosocial	Hard work Mindset Ask for feedback Communication Teamwork Consistency Improvement Competitiveness Player-coach relationship Grit Effort Lifestyle Aggressiveness Coachability	“[…] when you first come in, you've got to understand, you've got to look at the people who are seniors in the team, and understand the amount of hard work they put in to be where they are. And that you're going to have to put in easily that much, if not more, if you want to eventually get to that position. And if you can just get it in your head that it's going to take a hell of a lot of hard work to get into that position, then that's a good start. Because you get your first professional contract, that's just the beginning, you haven't even done anything yet at that stage. That's where all your hard work begins. So, the faster you can understand that, the faster you can start getting better”. PG2
	Technical-tactical	Position specific technical differences Good at basic drills Ball carrying Kicking skills for back Passing ball for width for forwards Decision making Big tackles Turnovers Great breakdowns	“You've got one or two things that you're really good at and some other stuff you're good at but it's not brilliant […]. Whereas in world class players, they make big tackles, make big carries, turnovers, work great at breakdowns, they've got everything”. PG1
Environmental constraints	National	Culture	“So, I started playing there, but yeah once again, all the way through secondary school probably did every single sport I could possibly do because it is how we do here [in England]”. PG2
	Socio-economic	Type of school	“If you really want to stick with rugby, it's kind of a private school thing…” PG1
	Family	Parents Brothers	“So, my dad was an actual ex-professional scrum-half as well so I used to do a lot with him. So, we’d go and that would be where I'd kind of get my basic pattern and kicking stuff done till I'd prepare myself to go and play in the academy in Wales”. PG1

### Task constraints

Task constraints consisted of three categories: (a) sport participation history, (b) activity type, and (c) game exposure. From a *sport participation history* perspective, it was evident that all players practiced different sports at various levels at a young age before or in concomitance with specialising in RU. For instance, a player from PG2 reported that:

“[…] But the biggest thing is making sure you do as many sports as possible because there's so many different skills that are transferable to the game of rugby […]. So, I think it's good to do as many sports as possible, just to increase your variety of skillsets.”

Moreover, a player from PG2 reported his diverse sporting background while focusing more specifically on RU:

“So, rugby is probably the main sport that I've really done. Done football, done kickboxing, done most sports, golf, but rugby's the main one”.

It also emerged that the *activity type* performed (e.g., in the form both of deliberate play and deliberate practice), was seen to have an advantageous effect on progression throughout a professional academy. As an example, it was reported that a larger accumulation of hours towards RU-specific activities was considered important for a player from PG1:

“I think mine was just definitely dedicating more time to it [training] than anyone else, really, when I was in those school kind of years. I was always kind of semi obsessed with going and getting passing and kicking done. In all my spare time it was just me and a couple of mates going to the local rugby club and kick for hours and hours. And it was our way of socialising as well through summers and stuff. So yeah, I think for me it was just kind of dedicating that time to go and develop my skills”.

Similarly, a player from PG2 reinforced the importance of engaging in additional RU-specific activities:

“So, you just find yourself doing extra gym sessions, extra kicking, like tackling with coaches and asking for more time and more feedback and stuff than like your peers around you who are not doing that”.

A player from PG1 reported that training similar in the form of deliberate play was what made the difference in his career:

“For me it was probably more enjoyment. When I was at school, I played it with my friends and we all went like training after school, which would be quite fun and we'd go back home. Then weekends, we'd play a game and then we'd go out for food or something afterwards and it was kind of like a fun activity to do […]. But for me, I think it was just making sure I kept enjoying it and didn't like overdo it and take it too seriously and ruin it for myself”.

The fact that the RU academy players had a large amount of *game exposure* was believed important for growth and development from a participant of PG2:

“So, I was playing like a Saturday, Sunday, sometimes on a Wednesday as well for the school. And when I look back on it and think you might say, ‘It was too much’. But I actually think it actually helped me a lot, because if you're tired and you physically don't feel as strong going into a game, it encourages you to challenge your skillset and do something in a different way”.

### Performer constraints

Performer constraints consisted of four categories: (a) anthropometric, (b) physiological, (c) psychological and psychosocial, and (d) technical-tactical. From an *anthropometric* viewpoint, a player from PG2 reported that being tall was a prerequisite to be a successful rugby player:

“[…] you’ve got to be people that are tall [to play professional rugby…]”.

Regarding *physiological* requirement important for the different game-positions in RU, a player from PG1 reported:

“[…] it's specific. It's not like football where, ‘Oh you'll pass, you'll shoot.’ Like the front row have just got to be big strong brutes, if you be a strong brute, you can [carry, attack and scrum], that's what you've got to bring. If you're a 9, you've got to bring your quick kicking game and your fast passing game. If you're a back, you've got to bring just speed in everything, over the top everything, just fast get in people's faces and stuff. I think you've got to bring that in your position and when you spend time with your mentor, you just learn off him”.

Players also require *psychological and psychosocial* characteristics, such as cohesiveness with the rest of the team, in order to progress and set apart from less successful academy players. For example, a player from PG2 reported:

“It's like the things that we're describing are sort of like the skeleton of the ideal player. But I feel like each individual person in that skeleton has got their own like flare or specific thing that they're good at. So, I feel like as a team we sort of fit into that skeleton by bringing our own like individual attributes. So, I feel like that's the best thing about being here, everyone has those individual attributes that just fit into that ideal player. So, I think there's no like specific, ‘He's the ideal player’. Everyone's got their own ability to become that ideal player, it's just fitting into that skeleton. […] It's more an ideal team”.

Moreover, results from the players' focus groups showed that several other *psychological and psychosocial* characteristics (e.g., hard work, communication, teamwork) had an important role in the TD process throughout the academy. These helped both in creating a successful teamwork environment and gaining trustworthiness. An example was provided from a player in PG1:

“If you're working hard and the guys around you know you're working hard they can trust you. And then if everyone's doing that together, then you'll work together well and it just makes a better team”.

The communication and the capacity to be resilient when asking for individual feedback was recognised by players to be a fundamental *psychological and psychosocial* characteristic both for the TID and TD process. For example, a player from PG mentioned:

“So, I think selection is a massive part of what we do in terms of like speaking to the coach and stuff, that's where you can get on their backs and say, ‘What do I need to do better to play? What can I improve? Where are my opportunities?’ that kind of thing”.

Mindset was also considered a *psychological and psychosocial* characteristic that differentiated standards of players. As an example, a player from PG1 stated:

“I think that's the difference between a really good club player and sort of the senior academy and making the first team. It's just that mentality”.

Another example confirming the importance of *psychological and psychosocial* characteristics in RU, was reported by a participant from PG2 when they stated that several other behavioural characteristics were considered beneficial to distinguish level of players (e.g., competitiveness, aggressiveness, grit):

“[…] And I think one thing that sets boys apart, so, from that jump where you go from academy to senior academy, you kind of see the boys that want it more. They go out on the field, they're more aggressive, they don't want to lose. So, when you've got some people that are just kind of there, they're good rugby players, they're talented, they're not going to make it because they don't have that extra little bit of grit”.

Regarding *technical-tactical* skills, a player from PG1 stated that practising basic RU drills was the prerequisite to be successful during the TID and TD process:

“If you don't have your basics [skills] then you don't have any chance, really”.

The connection between these four categories, which underscores the impact of a multitude of factors on the TID and TD processes in RU and the existing individual differences among players and positions, was explained by a player from PG1:

“I think around the table [focus group participants], like *player's name* sort of like brings a lot of physicality and work rate to games because he's like a very fit number 8. Like *player's name* brings a lot of speed like sort of around rucks and stuff like that and, like he says, with his kicking game. And *player's name* is just sort of like a wall in defence and like a strong runner. And personally, me, I like ball carrying, stuff like that is what I like to pride myself on”.

Another quote that supported the previous statement reported that every player has their own strengths relevant for the TID process. However, participants reported that for each player, there could be multiple important factors that could have influenced their personal progression across a professional academy, which was suggested by a player from PG1:

“But it's just everyone has their own individual thing that they're good at. There could be like two or three things, but I've just named one for each of us that I've seen in them. But there could be three or four, there isn't just one thing that you pride yourself on so there could be like a whole lot of various things”.

### Environmental constraints

Environmental constraints consisted of three categories: (a) national, (b) socio-economic, and (c) family. From a *national* outlook, a statement from a player from PG2 proposed how he may have played rugby due to his national sport tradition:

“But probably because rugby is the main sport in Wales as well, so everybody does rugby […] Because rugby's much bigger in Wales for kids than football […]”.

From a *socio-economic* perspective, in a sentence from a player in PG1 it was evident the clear impact of the sport orientation that different type of schools (e.g., private and state) could have on a RU player pathway:

“[…] Yeah well, not really [rugby] at state school, it was just football, everyone played football. Yeah, there was no rugby. It was literally the only sport you did; it was the only sport anyone did. And then, obviously, when I went to [private] school, it was just school rugby, nothing else. Unless it was cricket and so on but I hate cricket”.

*Family* was considered from PG2 one of the most important factors affecting progression in RU and in general in sports:

“Probably, a huge thing for I guess everyone round the country [England] would be family input because if you're raised up in a household of football then you're more than likely to be going into football and playing football more often. So, I was quite lucky in that sense because my family are massively into rugby and sort of like a wide range of different sports. So, they were open to me to play whatever sport I wanted to play and support me in whatever I wanted to do. And it turned out to be rugby and they were very happy about that. So, I think family is a huge thing for sportsmen to start off their career”.

A useful connection among these categories was highlighted throughout various statements. More specifically, a player from PG1 suggested:

“I went to a state school until Year 7 and then private school [in England] because all my brothers went there so I just followed the family. And then that's where I picked up rugby and started enjoying it”.

### Part two: coaches focus group

Table 4 reports the CG results and provides additional examples of relevant quotations for each of the categories identified and presented a complete overview of each sub-categories. Cumulatively, 34 sub-categories were found from the initial analysis of the raw data for CG. Further analysis revealed a total of eight categories perceived important by coaches for players’ TID and TD: (a) sport participation history, (b) game exposure, (c) anthropometric, (d) physiological, (e) psychological and psychosocial, (f) technical-tactical, (g) socio-economic, and (h) culture. Lastly, three themes represented the factors perceived to be determinant for CG on selection and progression of players in a professional RU academy. Three higher-order themes were consistent with the three constraints from the ecological dynamics framework (4, 9), based on the initial works of Newell (42), including task, performer, and environmental. As such, the proceeding results are presented using these higher-order themes.

**Table 4:** Coaches’ focus groups results.

Theme	Categories	Sub-categories	Categories’ additional example quotation
Task constraints	Sport participation history	Multisport background	“I think I'd just tell them to trust their instincts, try things they've learned from other sports, because so many of the young lads I have, they're trying to fit to a certain concept that they think is the right person to be and by doing so inhibit themselves quite a lot”.
	Game exposure	Amount of exposure to rugby play Exposure to play against older players	“I think it's good that we get them to compete against each other every now and again. So, we'll have the different centres come, and sometimes, if you're in your little bubble in your centre because at the younger [**0:36:50] centres, they get a sense that they might be very good. But actually, it's when you put yourself out there and you're playing against the other centres, that you realise that, actually, we've got some things to work on. And then as we go into those older age groups […], I know it's really bad. Let's say as under-16 s, we're going away to play other academy teams because, again, you get used to what your group is like but actually we need to see what that challenge is like, because, again, it's not just referencing the strength of our group, but it's referencing what it looks like nationally”.
Performer constraints	Anthropometric	Size Height Exclude late mature players	“I think, […], some of it is those behaviours, and [*coach's name*] mentioned as well, some of it is physical attributes. Basically, there are certain sized, shaped players that need to be in certain positions and if they don't have those attributes, it doesn't really matter how good their skillset is at that […]”.
	Physiological	Physical qualities over skillset Individual characteristics Fast	“So, again, it might be that Player A has good some really good skillsets, but Player B hasn't got them but he's got the physical attributes and size to be a premiership rugby player, so that's where we're going to have to put our resources”.
	Psychological and psychosocial	Hard work Work ethic Select players who ask feedback Commitment Select more coachable players Position specific requirements Slow processing players difficult to progress Mindset Behaviour Confidence Fearless	“When the ball's in the air and you're 50 m away from the ball but you're working as hard as you can to go and make a tackle or get a position to prevent to opposition scoring. Make a try-saving tackle or running 50 m to get an [offload] scrum, a score-winning try, whatever it is, but to see people work off the ball is a massive thing for the way I watch rugby and see behaviours within the children as well as the players”.
	Technical-tactical	Good at basics Sense of game Catch Pass Manipulate defence Footwork around the contact areas	“Do the simple things well. I think sometimes kids think that they've got to do the spectacular to showcase themselves, but actually just doing the basics really well [all the time]”.
Environmental constraints	Socio-economic	Type of school	“Actually being able to see every player play, whether it's a state school that plays [rugby] six weeks of the year and that's it or it's rugby clubs or big private schools, is having relationships with other schools and the guys out on the ground”.
	Culture	Positive and supportive environment Engagement with players Challenged but fun environment Quality of coaching Connection with local community Coaches teamwork Full-time environment	“I think, as *coach's name* touched on just now, it's the people that they have here. It's not as such just bringing in quantity of anyone with a rugby background and interest; it's making sure it's the quality and culture that comes in. So, everyone is here to work as part of that rugby family rather than individual interest just to try and beat each other”.

### Task constraints

Task constraints consisted of two categories: (a) sport participation history, and (b) game exposure. From a *sport participation history* perspective, one coach highlighted the importance of a multi-sport background:

“Just through my background and what I like to see in a player. If I see someone that plays [standoff or scrumhalf] in rugby, who's naturally a good footballer as an identified space from a young age, then you're like, ‘Yeah, we can work with this kid’. A lot of fly-halves that are at the standoff position in rugby have come through a football background. They will start with football and then be encouraged to see things and then move into rugby, and when you see them from an early age, they've usually got a good skillset, they've come from a background where they've been encouraged to work. And then it's just trying to give them more fine detail about the tactical stuff. And if they can take that on board, then usually you've got a bit of hope for them”.

Moreover, the connection between *game exposure* and developmental opportunities was explained by another coach:

“We do look at the scale of how much rugby they're playing week to week to keep it fair, when you're comparing them [for selection]”.

### Performer constraints

Performer consisted of four main categories: (a) anthropometric, (b) physiological, (c) psychological and psychosocial, and (d) technical-tactical. The CG highlighted the implications of *anthropometric* and long-term change upon TD outcomes. For instance, one coach reported that those players who did not mature physically in the time of 3 years would not progress across the academy:

“There are some kids who, through their athletic, their size, they haven't grown in a year or two or three, and they just won't fit into it naturally and we can't see any growth in them sometimes, physically, which is upsetting for them sometimes, but that's the way the modern game is going”.

From a *physiological* viewpoint, coaches reported that players' physical attributes have a key role during TID and TD processes. In fact, an example of statement confirming this concept is:

“Really just looking if a player's got an X factor. We talk about physical attributes. They might be really tall, you might have a guy who is really fast, they're not always the best rugby player but we might just have a look at someone athletically”.

The *psychological and psychosocial* area was also connected to TID and TD. In particular, coaches were unanimous on the topic of work ethic. As an example, one coach suggested:

“Just within that, once they come a bit older, it's also seeing a work ethic in them. So, when you're doing any hard work, and regardless if they're X factor or they're not, when you see a kid working, then it gives you a bit of hope that they'll learn and want to do better and they'd be competitive and they've got a work ethic to try and improve and get better […]. So, when you see the people graft and work hard, then you've probably got an eye for them as well when they stand out in bits and pieces that are not a glamorous part of the game. You realise that they've got an edge to them, and that usually stands out for me, which helps put them in a good place moving forward, as well”.

Confirming the importance of this *psychological and psychosocial* characteristics in young RU players, another coach specifically reported that the commitment to work hard was essential for successful players:

“So, […] is about having people who are really committed to working hard”.

Another *psychological and psychosocial characteristics* believed pivotal for a player to be selected and be able to progress across the academy was their capacity to ask for feedback and engage with training putting in practice information provided by coaches:

“[…] during the sessions, making sure that… little things like they're listening when we're talking to them as a group, making sure that they're paying attention, they're listening and maybe asking good questions and feedback around what we're trying to deliver to them. And for us, being able to see them trying to implement any points we're trying to work on with them. So, if we're looking at a certain skill, whatever it might be, seeing them actually trying to work on that and trying improve that when we then put them back into the game or the drill or whatever it might be; then seeing if they can, as the weeks go by, slowly take their individual points off everything and just keep adding to their game”.

On this basis, players with *psychological and psychosocial* deficiencies seemed to be less successful during the selection process, whereby a coach reported that:

“We also take into account […] learning difficulties and things like that. Because we've had kids who we don't necessarily think are listening in the summer [which is the period when U15 first selection take place], but then we've had a chat with them and we've realised that they're really dyslexic or they're slow in processing things. I think the most important thing is knowing your [player] that you're working with to boost the selection process”.

*Technical-tactical* factors were also an important element for TD. For instance, one of the coaches commented:

“We spend a lot of time on the 14 s, 15 s stage that we're at, the basics if you like, so being able to catch, pass well, manipulate defenders, footwork around the contact area, that sort of thing. Which hopefully then later in the years […] but how then we put that into, when the players get to more of the senior levels of the academy or into more of the first team area, whether they're still holding onto those points and hopefully adding to them as they go through, I would say”.

### Environmental constraints

Environmental constraints consisted of two categories: (a) socio-economic and (b) culture. An example of *socio-economic* factors, which references the type of school, is highlighted by the following statement of a coach:

“For example, when you compare a state school kid to a private school kid, the difference is how much rugby they play is very different. But that state school kid, you might be able to look at him and go, ‘Well, he's got more potential if we put a bit more rugby into him.’ So, we take that into account, and we look at where they go to school […]”.

The club's *culture* also appears to influence the TD process. For instance, one coach reported:

“I think the whole culture of *club's name* adds to it [referred to the TD path] massively […]. But it's that whole culture of there's a friendship and quality there, so you feel comfortable, and you feel comfortable in whatever you do, which adds to the strength of what we're trying to achieve in the bigger picture”.

Furthermore, another coach suggested the importance of creating a developmentally appropriate *culture* for young players to flourish:

“the environments we have for them are challenging but actually are fun environments, such as the coaching groups that we have within the pathway”.

Additionally, another coach provided a similar sentiment to reiterate the importance of club culture and quality of coaches in developing young RU players:

“[…] it's the people that they have here. It's not as such just bringing in quantity of anyone with a rugby background and interest; it's making sure it's the quality that comes in […]”.

**Figure 1:**
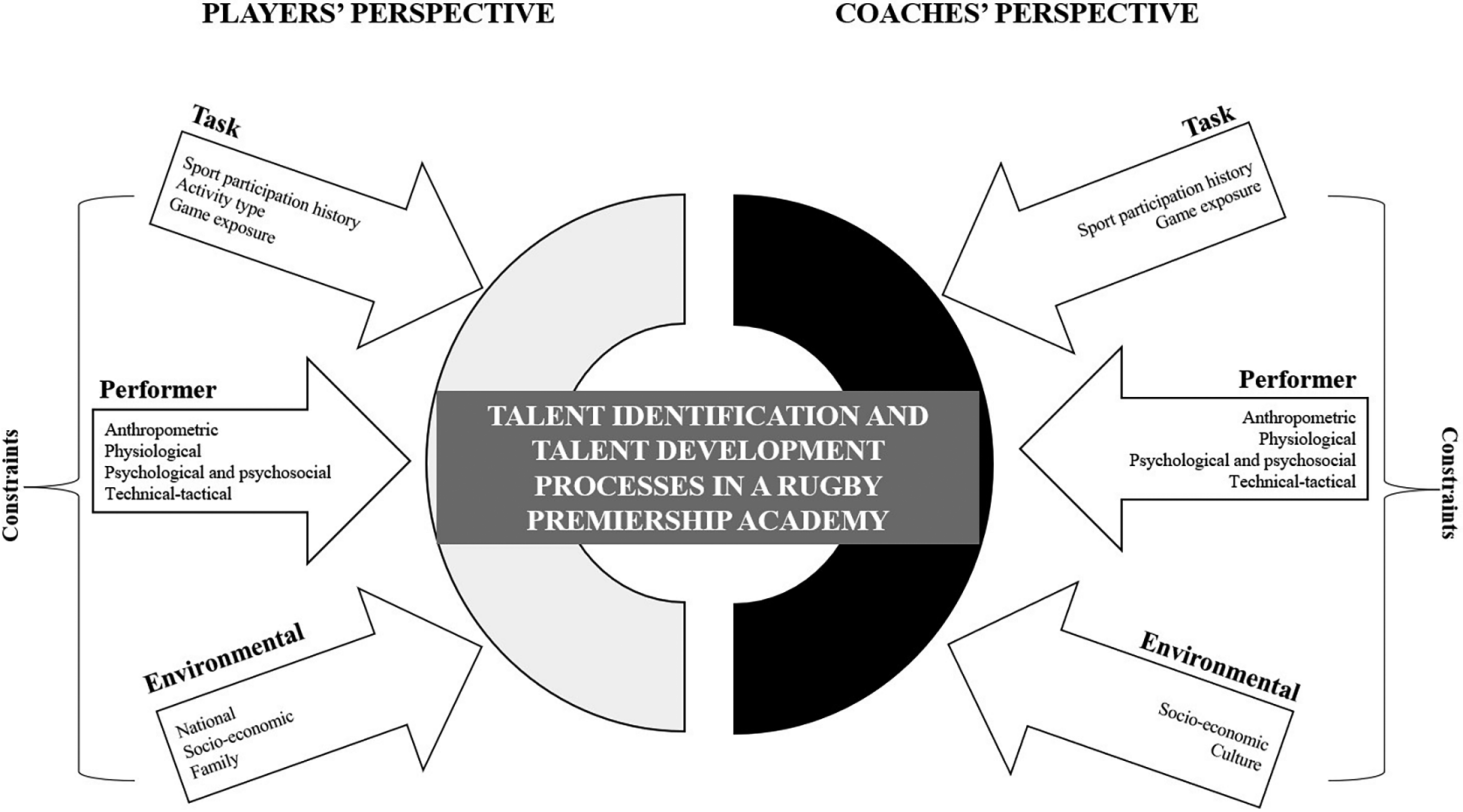
Summary of players’ and coaches’ perspective on TID and TD process in a premiership RU academy.

Interestingly, a statement from a coach outlined the connection among the high-order themes emerged:

“We've just offered some academy players contracts, and a couple of them have been given contracts just because of the size of them, and we think that they're going to grow. We're hoping that they're going to grow, being in a professional environment. So, they've been at school every day, and they've not had a chance to go to gym every day. They're restricted to how much training they can do, and hopefully by bringing them into this environment, they will grow physically as well as athletically and they'll fit into that” (Figure 1).

## Discussion

The aim of this qualitative study was to explore the perceptions of the TID and TD processes in senior academy players (part one) and academy coaches (part two) from an English Premiership RU club. Findings revealed that despite inconsistencies in some of the factors compounding categories and sub-categories, both players and coaches perceived task, performer, and environmental constraints to be important aspects of players progression. Previous studies indicated that, although not always analysed in unison, these three areas were the most researched in different countries during the TID and TD processes in RU (9). These constraints are part of a “*constraints-led approach*” and are understood as boundaries that shape the path (52) and emergent actions of athletes (42). Broadly, constraints are classified into one of three classes: task, performer, and environmental (42). Task constraints typically relate to the sport activity performed by athletes in time. Example of these include game played, training methods, and exposure to certain training stimuli. Performer constraints pertain to characteristics of an individual, like anthropometrics, physiological qualities, emotional and psychological states and arousal levels. Environmental constraints include contextual aspects external to the performer, such as ambient, training availability, socio-economic status, quality of coaching, culture and social support. Overall, findings of present work reinforces the suggestion that a holistic approach is required when identifying and developing talents in RU (39).

Despite differences amongst players' and coaches' groups, *task constraints* were expressed as important aspects across both cohorts. In fact, all participants suggested that sport participation history and game exposure were characteristics that would discriminate players progressions across a RU talent path since, as found in previous studies, these aspects facilitated skill acquisition, skill transfer, and were decisive in reaching high performance status (53, 54). Whereas diverse opinions were found among players on the impact of the extra type of activities (e.g., engagement in RU or multisport activities) on TID and TD processes. Interestingly, the majority of the participants stated that the dedication, and sometimes the “obsession”, for performing additional, repetitive drills following scheduled training was believed an important part of the self-improvement process. Only one player had the opinion that, to avoid burnout, he preferred to be involved in extra training that he perceived enjoyable.

These concepts recall the existing activity framework in sport and training that indicate that both *deliberate practice* and *deliberate play* are important aspect of skill acquisition and athlete progression in sports (55) and provide practical recommendations to RU coaches. Deliberate practice has been defined as a *coach-led* activity characterised by frequent repetitions and corrections of a task near or beyond the athlete's current capability, and is physically and mentally highly effortful and not inherently enjoyable. In this type of activity, athletes may perceive enjoyment from the anticipated improved performance, but not from the practice activity itself (56). Deliberate play, instead, has been defined as *peer-led* activity characterised by involvement of athletes in informal sports play with peers with the purpose of maximizing inherent enjoyment in the activity itself rather than improving performance [see the Developmental Model of Sport Participation for a deeper analysis; (57)].

The present findings are in line with a recent multi-disciplinary work on an English Premiership RU academy, which demonstrated that greater exposure to different activities during childhood and early adolescence had a positive impact on higher player rankings (58). Curiously, coaches did not argument the possibility that some activities could be better than others (i.e., deliberate play vs. deliberate practice) for players progression, thus it seems that this factor do not impact significantly upon the talent path. Present findings suggest that: (a) a varied learning experiences could facilitate rugby-specific skill acquisition (59), and enhance general athletes functionality (60), (b) practitioners should understand the pathways young players have taken during the TID process, (c) integrate both types of activities (e.g., deliberate play and deliberate practice) during all phases of development, and (d) a better communication should be used by coaches surrounding both players expectations and the key parameters useful for players' progression in the current academy.

*Performer constraints* was one of the present themes of the qualitative analysis. All participants highlighted those factors related to anthropometric, physical, psychological and psychosocial, and technical-tactical skills were integral to TID and TD processes. Body height was an anthropometrical parameter recognised necessary for a successful RU performer by both players groups. However, although literature has reported that this variable has some degrees of importance in RU (61, 62), it has not been recognised significantly impactful for progression as players' body mass (63). Importantly, the coaches reported that one of the essential aspects for players to be selected was that, over every other performance factor, players should possess specific “body sizes”. Unsurprisingly, they reported that late maturing players had more difficulties in progressing across a RU academy environment, confirming the fact that maturation status could bias selection of future talents in RU (3). This indicates that coaches generally recognised the importance of anthropometric measures in RU. Thus, present results suggest that (a) anthropometric measures (e.g., height and body mass; referred as *size*) along with (b) players' maturation status, should be carefully monitored during TID and TD assessments in order to observe progression and avoid potential bias.

Position-specific physiological traits were considered important for players' progression from both players and coaches. While players outlined that, despite the position individual characteristics of a player, a multitude of physical aspects (e.g., speed, strength, fitness level) contribute to players’ success, coaches indicated that physicality was the most important attribute (even more than RU skills) to distinguish young talents in RU. These findings are in line with recent research on regional English (12, 13, 64) and Italian RU academies (65), which consolidates the understanding that specific physical variables are based on positional roles and can determine the successful progression of RU players across an academy. Overall, present physiological findings further inform the research field that physicality (e.g., anthropometric and physiological qualities) are more predictive of selection when compared to other qualities in RU.

Both players and coaches believed psychological and psychosocial characteristics to be part of a holistic set of pivotal characteristics for TID and TD in RU, which aligns with recent findings in sport psychology (11, 58, 66). In particular, the recognised capacity of working hard (from PG1, PG2, and CG), having the right mindset (from PG1 and PG2), possessing a strong work ethic (i.e., a set of values centred on the importance of doing work and reflected especially in a desire or determination to work hard; from CG only), and the capacity of asking for feedback (from PG1, PG2, and CG) were viewed as fundamental behavioural characteristics to become a professional RU player in the present academy. Moreover, similar psychological traits emerged from the analysis of the behavioural characteristics considered important by coaches and staff in English (17) and Zimbabwean (16) RU environments (e.g., capacity of working hard, possessing the right mindset in- and out-game situations, the ability to communicate effectively, and the skillset to display an high level of resilience during critical situations). In particular, in the present work, among all psychological and psychosocial characteristics, the concept of *hard work* (i.e., the ability of a player to be constantly, regularly, or habitually engaged in working hard toward a pre-set objective) was the most cited by both cohorts, indicating that, in line with previous findings in sports (67), the persistence and dedication to “do extras” to become a better player was one of the most important qualities a players should possess to reach professional status. However, this characteristic should be monitored by coaches and players since it could represent a possible trigger of player burnout in RU (68). In conclusion, results on the psychological and psychosocial characteristics that are perceived by players and coaches as important to becoming a professional player indicated that individualised sport psychology programmes should be: (a) incorporated to assess and help develop these characteristics in young players, (b) regularly structured across RU academies to optimise players progression, and (c) focussed in implementing the players capacity of working hard.

Technical-tactical attributes were considered an important parameter for TID and TD in RU. For this category, while both players focus groups reinforced the concept that each playing position has its own technical-tactical requirements, they also highlighted that player's success is relative to how successful groups of RU players play together rather than individually [i.e., tactical playing cohesion and collective effectiveness; (69)]. In contrast, coaches suggested that when assessing technical-tactical competencies during TID and TD, their evaluation focused on players’ capacity to perform the basic RU drills well (e.g., passing, catching, kicking, tackling). It has previously been reported that basic technical drills discriminate levels of RU athletes in schoolboy (70), and academy players (71) in RU, thus confirming the importance that basic skills have during players' progression. Altogether, present findings suggest that: (a) players must develop basic technical drills, (b) players and coaches should consider the importance of tactical collective effectiveness, (c) coaches are encouraged to include a range of technical and tactical activities into their session design to achieve these outcomes, and (d) coaches should be clearer on which technical and tactical aspects they believe important for player to develop.

Regarding the *environmental constraints*, participants of this current study recognised national, socio-economic, family, and culture as vital aspects for players progression. However, only one factor (i.e., socio-economic) were believed important from both groups of participants. Interviewed players believed that one of the reasons for an initial successful identification and development in RU was the nation of provenience, since an elevated popularity of RU in the area of origin could offer more chance of initiation and continuous engagement. This concept is in agreement with Winn et al. (72), which reported that, despite the impact of social deprivation, an important effect on RU players' career in Wales, initial youngsters' participation in recreational non-controlled RU activities was still high in areas far from adult-led environments. This phenomenon was due to the elevated popularity of RU across all local communities in Welsh provinces. Similarly, Marsters and Tiatia-Seath (73), documented how both RU and rugby league were activities largely entrenched in most poor Pacific Island communities, which could have influenced the tendency of young Pacific Island players to pursue a career in either type of rugby code. Thus, the results of the present work indicate that the level of popularity of a sport in a country could affect the trajectory of talented players. Therefore, RU coaches operating in nations where RU is not of a major national interest, should implement meticulous and effective strategies to optimise TID and TD operations.

Socio-economic status was the only category among the environmental constraint believed important both from players and coaches. In particular, all the examined groups differentiated the aspect of players attending private and state schools, indicating that those athletes deriving from private schools had several more chances to progress in RU than those who studied in state schools, which is due to the different investment of the two types of institutions in this sport. Previous research supported this concept, reporting that both in the context of RU and rugby league, private schools in the South-Eastern Hemisphere (i.e., Australia and Pacific Islands), were often considered “better schools” and were more oriented to either form of rugby training than state schools placed in poorer areas of the country (73–75). Present results lead to some practical suggestions that should be considered with caution, including: (a) managers of professional RU academies should include a socio-economic assessment when initially selecting RU players in order to avoid TID bias linked to type of school of provenience, and (b) attempt to develop partnerships with local state schools in order to avoid missing potential talents.

Family support was seen as an important factor from both player groups. Specifically, they suggested that both parents and brothers provided the right motivation, attitude, and economic support to engage in RU activities. This result was in line with TID and TD literature in sport (76), whereby it has been shown that family (i.e., parents and siblings) had a strong impact on player's sport initiation, engagement in activities, and consequent progression. Similarly, research on Pacific Islands players from both RU and rugby league codes reported that players considered their families a source of support to remain grounded, focused, motivate them to self-improve (73), and represented a decisive financial aid in their development (75). Moreover, this finding add value to the existing ecological dynamic framework theory applied to RU (9, 12, 13) since it indicates, for the first time in this sport, the important impact that family has on players' progression in the specific code of RU based on both player and coach perceptions. Therefore, RU organisations and practitioner should educate relatives to ensure they are aware of the important role they play in the life of young RU players, since emotional and economical support could impact significantly on players' career.

According to coaches, the culture surrounding the academy environment of a Premiership RU club is a fundamental aspect for players' growth. The interviewed members of staff mentioned that a challenging, positive, and supportive full-time RU academy environment was the key for optimal TD. However, in the coaches' opinion, these environments could only be created by clubs who recruit high-quality coaches. In fact, the importance of the appropriateness of personnel in elite sport academies has been already reported by several authors investigating the TID and TD processes. For example, in order to potentiate the athletic development of an athlete, Lloyd et al. (77) reported that personnel aiming to work in professional academies should possess an appropriate understanding of technical aspects of training (e.g., strength and conditioning knowledge), relevant working experience in the field and an appropriate qualification path. In this view, coaches could be considered the “architects” of the performance environment (78), and thus they should possess both the intra- and inter-personal skills (e.g., effectiveness and experience) to face the responsibilities required in an elite long-term athlete development environment (79). Therefore, these results suggest both that managers should focus on their staffs' coaching effectiveness [i.e., interpersonal, intrapersonal, and professional skills; (80)] to create an effective RU environment, and coaches should reinforce the importance of *culture* to all-age players during their path towards the senior professional status to improve motivation in their athletes.

### Limitations

A limitation of this study is the number of participants recruited. A similar number of individuals has previously been recommended for focus groups (21), yet a higher number of participants in the present investigation may have decreased discrepancies within groups findings, as well as potentially added additional findings or inconsistency in disagreements. It is also important to mention that although all coaches were qualified according to RFU criteria, and players regularly followed club's theoretical developmental workshops surrounding sport sciences, their knowledge of the scientific terminology regarding anthropometric, physiological, and psychological and psychosocial characteristics (e.g., “size”, “fitness level”, “hard work”, “work ethic”, “mentality” and “mindset”) could have been used inappropriately during focus groups discussions. Importantly, it should be mentioned that despite players reported information on their weekly competition routine (e.g., number of games played in two or three consecutive days), the authors do not necessarily intend to promote as much game exposure as referred in a PG's quote due to the potential risk of physical and psychological injuries many consecutive matches could lead to. Moreover, due to the novelty of this manuscript, comparison with similar investigations in RU was difficult, therefore it was not possible for authors to make further connections with other qualitative studies in this sport that could have better highlighted participants' positions in regards of TID and TD processes. This aspect represented a constraint that limits the ecological validity of present findings. It is also worth considering that RU players and coaches of different countries may have different perceptions on TID and TD paths, since the understanding, vision and philosophy of the game may change depending upon social context. In addition, players' age and playing position could have addressed focus groups' discussion towards themes that would not reflect necessarily the opinion of younger academy players, therefore different voices could have led the present research to different conclusions. It is also worth to mention that similarities in some of the results, final themes, and in some of the categories, could have been due to the homogeneity of the cohorts used in the current study (e.g., they belonged to the same club playing in the English top RU division and therefore, their beliefs could have been affected by the club's trend). Another important limitation is the one regarding the research approach used. In fact, while a cross-sectional analysis provided an immediate insight into the vision of an English Premiership RU academy, a longitudinal investigation on this topic could consolidate the validity of present findings. In this light, more qualitative investigations in academies of professional RU clubs are needed.

## Conclusion

This is the first published study that has analysed the perceptions of senior academy players and academy coaches on the TID and TD processes in an English Premiership RU club. These preliminary findings demonstrate that task, performer, and environmental constraints were the parameters considered important during the players journey towards senior professional status. Despite results aligning to previous holistic findings (9), and adding knowledge to existing theories on ecological dynamic framework in RU (e.g., revealing the important role of family in RU), there was an equal emphasis from both players and coaches on the impact of performer constraints on TID and TD paths. Whereas the difference in task and environmental constraints showed some inconsistencies among the two populations of this study, indicating that despite commonalities in final themes, perspectives of players and coaches on TID and TD were controversial on several aspects. This could reveal important implications on the TID and TD approach in English RU academies, since it appears that players are not always aware of all factors that coaches believe are important for the selection and development processes. Therefore, although more qualitative research is required in this population, the present study could be used by practitioners as a guideline to optimize a multidisciplinary approach to TID and TD in RU. In practical terms, this investigation can be resourceful for practitioners to shape an optimal developmental environment in the academy of a professional RU club (e.g., employing qualified coaches which align to the club's culture, incentivising educational moments for players' parents, and promoting partnerships with both private and public schools). Moreover, the implementation of these recommendations will be key both in ensuring that players become more aware of the holistic requirements needed during TID and TD in a professional academy, and coaches adequately support athletes in pursuing the journey towards the senior professional status.

## Data Availability

The original contributions presented in the study are included in the article/Supplementary Material, further inquiries can be directed to the corresponding author.
